# Supplementary material for: Fumed Silica-Based Ultra-High-Purity Synthetic Quartz Powder via Sol–Gel Process for Advanced Semiconductor Process beyond Design Rule of 3 nm
Source: Nanomaterials (Basel). 2023 Jan 18;13(3):390. doi: 10.3390/nano13030390 (PMC9919415; doi:10.3390/nano13030390)
Supplement: Supplementary file 1 [file nanomaterials-13-00390-s001.zip › nanomaterials-2154805-supplementary.pdf]

nanomaterials

MDPI

Article

Fumed Silica-Based Ultra-High-Purity Synthetic Quartz Powder via Sol–Gel Process for Advanced Semiconductor Process beyond Design Rule of 3 nm

Ji-Ho Choi [1], Woo-Guk Lee [2], Tae-Hun Shim [3], and Jea-Gun Park [1,2,3,*]

[1] Department of Electronic Engineering, Hanyang University, Seoul 04763, Republic of Korea
[2] Department of Nanoscale Semiconductor Engineering, Hanyang University, Seoul 04763, Republic of Korea
[3] Advanced Semiconductor Materials & Device Development Center, Hanyang University, Seoul 04763, Republic of Korea
* Correspondence: parkjgl@hanyang.ac.kr

Table S1. Comparison of purity level for amorphous silica particles and synthetic quartz powder.

Scheme 40.	Raw material	Method	Purity level	Ref.
Silica nanoparticle	Tetraethyl orthosilicate (TEOS)	Sol–gel	-	[40]
High purity silica	Commercial waterglass	Sol–gel	99.95 wt%	[41]
High purity spherical silica nanoparticles	Natural quartz powder	Sol–gel	99.92 wt%	[42]
High purity amorphous silica	Oil palm fond	Sol–gel	Above 90%	[43]
High purity amorphous silica	Rice husk	Sol–gel	Above 95%	[44]
Synthetic quartz powder	Fumed silica	Sol–gel (via ion exchange)	99.99999% (impurity level: 74.1 ppb)	This work

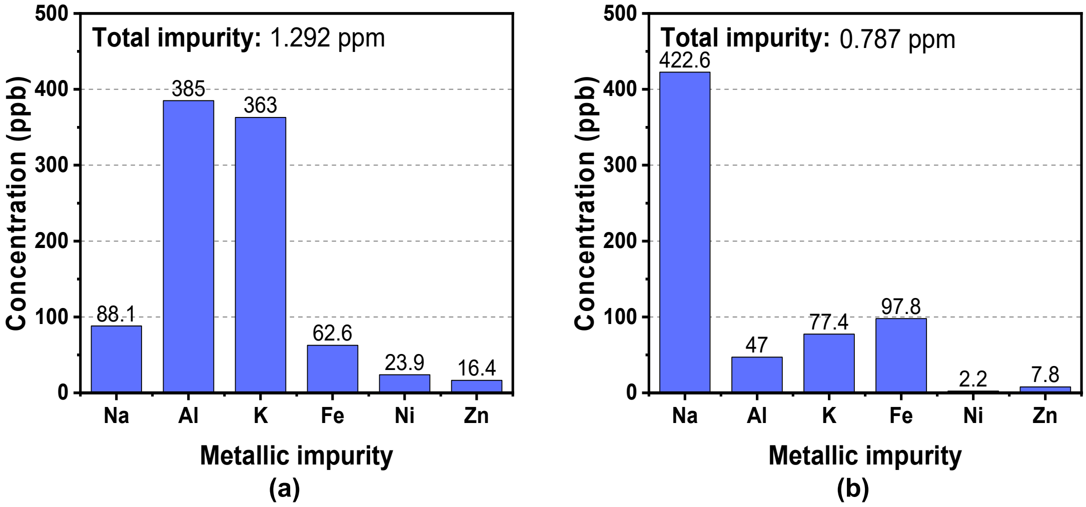

Figure S1. Metallic impurity concentration of (**a**) fumed silica and (**b**) colloidal silica.

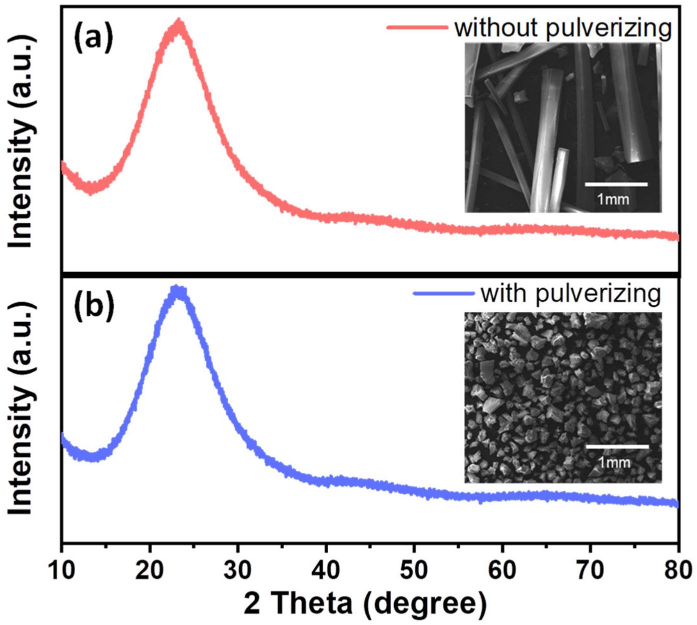

Figure S2. Effect of pulverizing process on quartz powder crystallinity, analyzed by XRD (**a**) without pulverizing process and (**b**) with pulverizing process, where background SEM images present the rod-shaped powder without pulverizing process and the granular-shaped powder with pulverizing process.

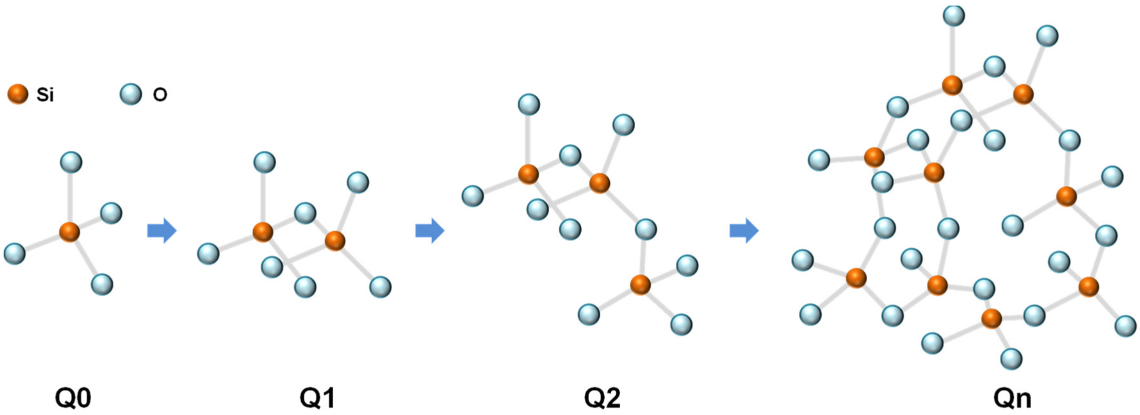

Figure S3. Mechanism of silica polymerization based on polycondensation of monomers during gelation process.

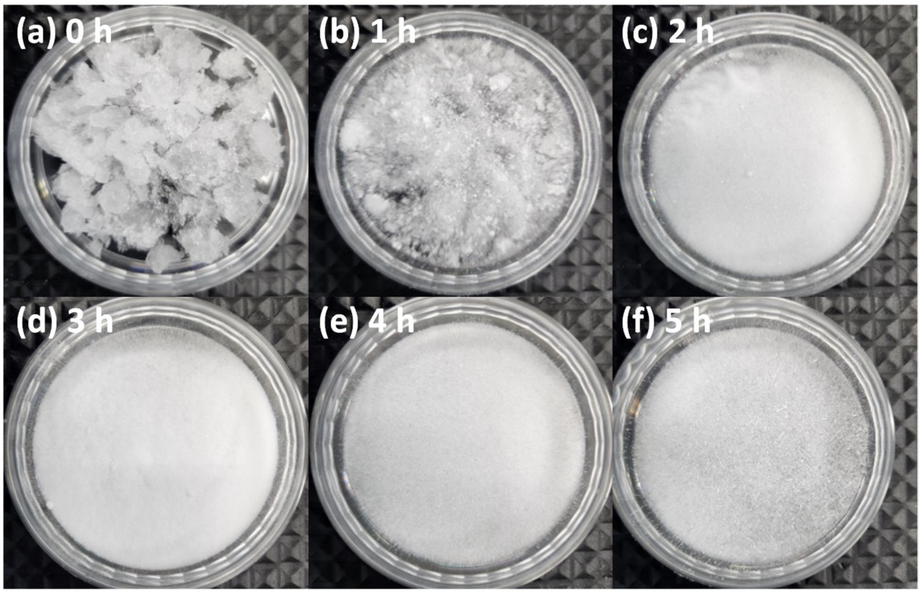

Figure S4. Photograph images of as-synthesized quartz powder with varying gelation time ranging from 0 to 5 h.
